# An interview with Feng Shao and Weihong Tan: chemistry intertwines with biology and medicine

**DOI:** 10.1093/nsr/nwz165

**Published:** 2019-11-11

**Authors:** Weijie Zhao

**Affiliations:** NSR news editor based, Beijing

## Abstract

Professor Feng Shao at National Institute of Biological Sciences, Beijing is a well-known immunologist. He has made a series of fundamental contributions to the fields of pathogen-host interactions and innate immune response mechanisms. Professor Weihong Tan at Hunan University and Shanghai Jiaotong University is a world-renowned scholar in bioanalytical chemistry and chemical biology. He developed a number of chemical tools that expended the extent of molecular medicine and may turn into useful clinical diagnosis and treatment tools. In May 2019, National Science Review (NSR) had an interview with Professors Feng Shao and Weihong Tan. The two scientists, both trained at University of Michigan for their PhD study, have different research directions, but share the same basic scientific vision: to solve human health-related fundamental problems by illuminating and making use of the underlying molecular mechanisms. They also share similar opinions on biomedical research methods and scientific career development.

## CHEMISTRY PROVIDE CONCEPTS AND METHODS FOR BIOMEDICAL SCIENCE

**NSR:** You both majored in chemistry as undergraduates, and now perform research works related to biology and medicine. What is the importance of chemistry to biomedical science?

**Shao:** On one hand, chemistry brought the concept of ‘mechanism’ into life science. Today, life science and chemistry both aim to understand the mechanisms of biological processes or chemical reactions at the molecular level. Bio-molecules such as nucleic acids, proteins and lipids are all chemicals, so if we want to understand the mechanisms of biological processes, we have to work at the chemical and molecular levels.

On the other hand, biological research largely relies on the methods and technologies supplied by chemistry. Biology itself only has generated two main research methods: molecular biology methods such as gene editing and antigen-antibody recognition-based methods such as immunohistochemistry. In addition to these two types of methods, life science uses a large number of methods provided by chemistry, physics and other research disciplines. Chemical probes, chemical intervention reagents and other chemical tools have greatly benefited life science research.

**Tan:** I am a molecular scientist and always perceive the world from a molecular point of view. I consider the human body as a molecular machine and believe that every biological process can be explained by its molecular basis. When somebody is ill, it is certain that we can find the cause of illness within the human molecular machine, i.e., some abnormality or aberration of molecular interactions. The first step to understand, diagnose and treat disease is to illuminate the underlying molecular mechanisms.

And as Shao said, chemistry provides many molecular tools for biomedical research. No tools, no science and development!

**Figure 1:**
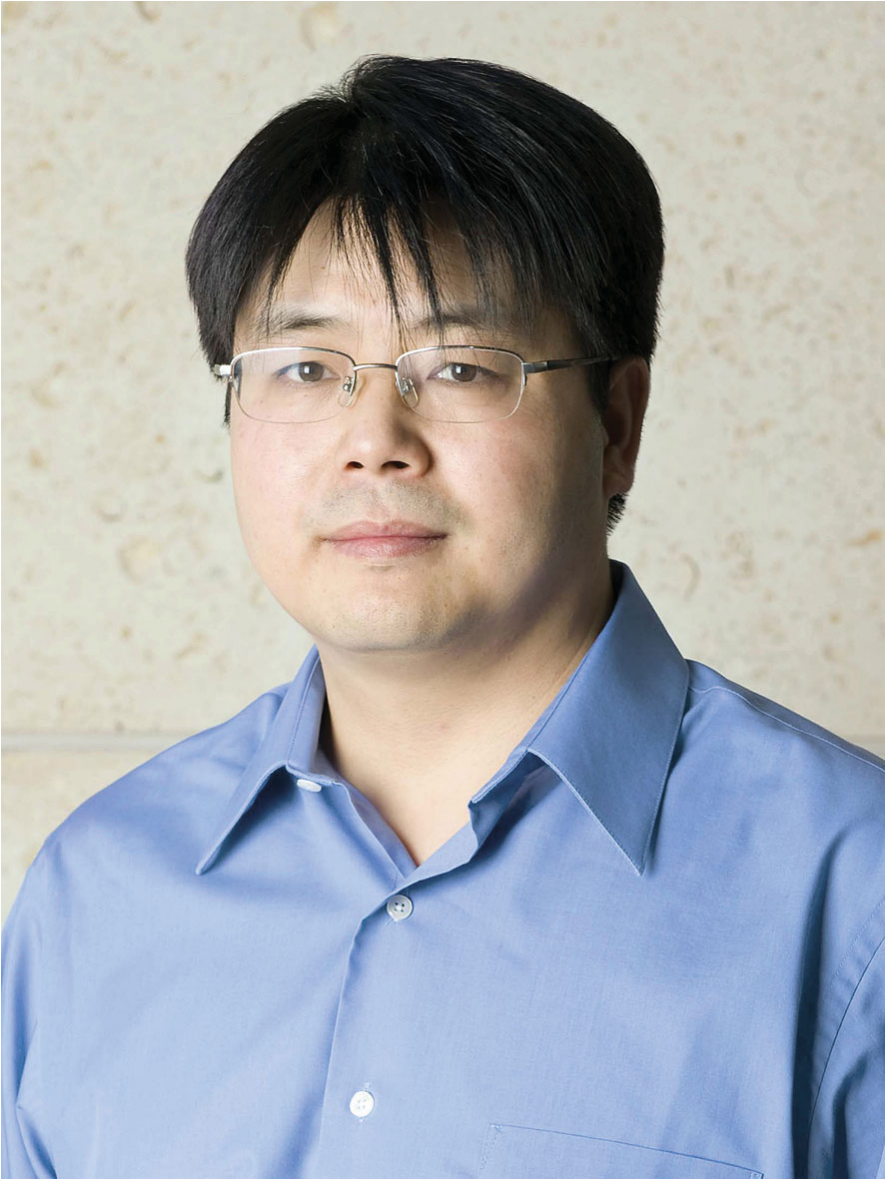
Professor Feng Shao is an immunologist focusing on pathogen-host interactions and innate immune response mechanisms *(Courtesy of Feng Shao)*.

Chemistry is a relatively mature discipline compared to biology and medical sciences. There is a Chinese saying that ‘If you want to do something well, you must first sharpen the tool.’ If you want to perform high level biomedical research and discover cures for disease, you need to have a deep understanding of molecular science and exploit all available molecular tools. Chemical ideas and tools will propel the development of molecular medicine.

## THE EVOLUTIONARY FIGHT BETWEEN PATHOGEN AND HOST

**NSR:** What are the major facts about the interaction between pathogens and hosts we have learned in the past 20 years? What are the things still not known?

**Shao:** The relationship between pathogens and hosts can be considered as an evolutionary fight. For instance, there is a layer of lipopolysaccharides (LPS) on the surface of Gram-negative bacteria, which can be recognized by specific immune receptors in host cells; to cope with this challenge, the bacteria, when present in the host may modify its LPS structure to avoid host immune recognition; to ensure recognition of the bacteria, the host in turn may evolve an additional set of molecular machinery to recognize other conserved molecules from the bacteria.

There are basically two things that pathogens often want to do: the first is to escape, inhibit and block the immune recognition and immune clearance by the host cells; the second is to establish a safe niche in the host by altering the normal physiological processes and states of the host cells, which may lead to a series of pathological changes. Pathogens often accomplish these missions by using molecular machines and the secreted toxins.

On the other end of the evolutionary fight, hosts want to antagonize these pathogenic processes. They recognize conserved molecules or virulence activity associated with the pathogens and then activate innate immune responses typically by secreting inflammatory cytokines and undergo inflammatory cell death such as pyroptosis. These defense responses can limit pathogen survival directly or more often call other parts of the immune system come in to clear the infectious agents.

In the past decades, we have significant progresses on both sides of the evolutionary fight. At the pathogen side, we have learned a lot about the mechanisms and effects of many important bacterial toxins and effectors, and on the other side, we have discovered many host immune receptors that are dedicated to recognize those bacteria-derived signals.

Despite all these, we are still far from understanding the virulence mechanism in full for many medically important bacterial pathogens such as *Mycobacterium tuberculosis*. Our researches about caspase-11 as the intracellular receptor for LPS and identification of the downstream pyroptosis executioner protein gasdermin-D (GSDMD) have solved the puzzle about the Gram-negative bacteria-induced septic shock. However, we still know little about sepsis caused by Gram-positive bacteria and fungus, which presumably should also be caused by overactivation of the anti-bacterial inflammatory responses.

In innate immune recognition of bacteria, since the first discovery of the Toll-like receptor for extracellular LPS (which was awarded with the Nobel Prize in 2011), we have contributed significantly in cytosolic recognition of bacterial products or activities. Given that the space or spectrum of bacterial pathogens that mammals may encounter is huge, it is conceivable that there should be many more cytosolic innate immune receptors that have not been discovered. My group along with many others worldwide are actively pursuing towards this direction. I am positive that there will be more exciting novel discoveries coming out in the coming decade.

**Figure 2:**
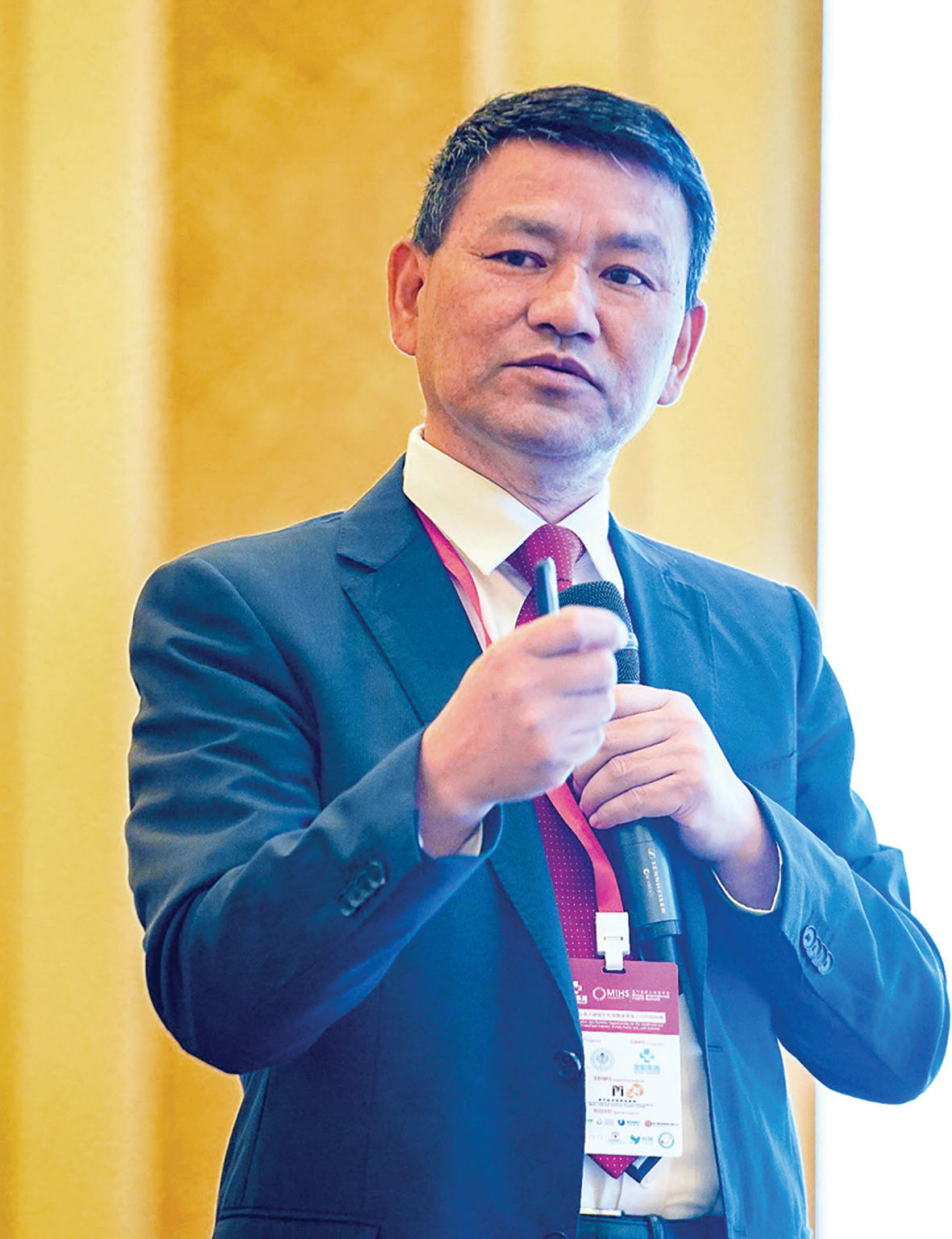
Professor Weihong Tan is a scientist of bioanalytical chemistry and chemical biology. Now he is mainly working in the field of molecular medicine *(Courtesy of Weihong Tan)*.

Finally, we are also facing a big challenge in understanding the anti-bacterial immune responses and their pathological significance at the system level in the animals. We know that a particular cytokine like interferon may have an important function under a given context. We also know that neutrophils are important in clearing certain bacterial infection. But are there other inflammatory mechanisms effective in fighting against bacterial infection? How do all different immune responses coordinate and function together in helping us to defeat a particular deadly infection? All these questions are the subject of study in the next few years.

**NSR:** Are there commonalities among the infection mechanisms of different pathogens?

**Shao:** Pathogen is quite a broad concept which includes viruses, fungi and bacteria. For bacterial pathogens, there are certainly common virulence strategies among different bacteria, but each bacterial pathogen also has its own unique mechanism. As for immune recognition, bacteria are a lot more complex than viruses given their larger genome and more complicated structures. Following immune recognition of different bacteria or pathogens, there are also common modes of defenses, including the inflammatory responses. For example, detection of different bacterial pathogens may lead to a similar cytokine response or host cell pyroptosis.

**NSR:** Your research works were relatively basic. Do you have plans to go closer to the clinic?

**Shao:** For our research on bacterial virulence mechanism, what we have been studying is mostly model pathogens such as *Salmonella*, *Shigella* and pathogenic *Escherichia coli*. For antibiotics or anti-infection agent development, it is not wise or practical to target the unique virulence mechanism that we have learned from our basic research on a particular bacterium as the industry is clearly more interested in having effective vaccines against those bugs. In fact, our research on bacterial virulence may provide some insights or clues into vaccine development, but we have not moved into that direction because vaccine development is not something particularly interesting to me. So in this area, the primary goal of our research is to learn new knowledge and make novel scientific discoveries.

On the other hand, our research on innate immunity and pyroptosis seems to be much closer to be potentially translated into something useful in clinics. In innate immune recognition, we have discovered several bacteria-derived molecules, like ADP-heptose, that can promote inflammation in mammals. These agents are potentially useful to be developed into novel immune modulators, for example in promoting anti-cancer immunity. Our discoveries on gasdermin and pyroptosis also provide novel targets to generate anti-inflammatory agents for diseases like sepsis or other diseases caused by hyperinflammatory responses. We are encouraged by the fact that genetic mutations in the inflammasome and pyroptosis pathway have strong associations with autoinflammatory or autoimmune diseases. Interestingly, recent studies suggest that an agonist for pyroptosis induction apparently holds the promise for improving cancer immunotherapy. Along this line, we are actively collaborating with the industry to move more towards translational development. We are also doing some translational studies in our own lab, hoping to obtain something of real and immediate intellectual properties for drug development. We will see how these efforts will pan out in the next few years and then decide whether we should set up a biotech company to translate our original discoveries. In this regard, we will definitely need complementary expertise such as in medicinal chemistry and pharmacology.

We are still far from understanding the virulence mechanism in full for many medically important bacterial pathogens such as *Mycobacterium tuberculosis*.

—Feng Shao

## FROM BASIC RESEARCH TO CLINICAL APPLICATION

**NSR:** Your (Tan's) group has developed a number of DNA based molecular tools. Why are you interested in DNA molecules?

**Tan:** Endogenous DNAs are traditionally considered to be genetic materials. But nowadays, scientists have designed and synthesized many functional DNAs, which are usually DNA molecules shorter than 100 bases or base pairs. For example, we can synthesize a kind of short DNA molecule with designed sequences able to recognize certain antigens, performing more or less like antibodies. We call them aptamers. Then, we can use chemical methods to further optimize these molecules and make them more stable, more selective and more suitable for *in vivo* applications, thus transforming them into diverse kinds of clinically applicable molecular recognition probes.

**NSR:** Are these molecules under clinical trials?

**Tan:** We are performing several clinical trials in cooperation with hospitals such as Renji Hospital of Shanghai Jiao Tong University, Xiangya Hospital of Central South University and the Cancer Hospital of the University of Chinese Academy of Sciences, Hangzhou. But as you know, the success rate of clinical trials is rather low. So, I cannot definitely tell when our tools can be applied in hospitals. However, we have four aptamer drug conjugates (ApDC) developed and at the early stages of clinical trials.

**NSR:** In China, are there any obstacles for the transformation from basic research to clinical applications? Are there any unstandardized processes?

**Tan:** The China Food and Drug Administration (CFDA) is less experienced in new drug approval compared to the US FDA, so the CFDA used to be very cautious. It would only approve new drugs after similar drugs are approved in the US. But we can see that China is paying much attention to new drug discovery today. Our transformation process is becoming more and more standardized. I believe that China's drug discovery system is poised to develop first-in-class drugs in the next 10 years in a manner comparable to that of the US.

For instance, China set up a Science and Technology Innovation Board within the Shanghai Stock Exchange. Biomedical startups can be listed on the Science and Technology Innovation Board during a relatively early stage of development. China is making big efforts toward transferring part of the money we have put into real estate and other areas to different fields of science and technology, including drug discovery and new diagnostic technology development.

The Chinese Academy of Sciences (CAS) has just established its first institute in medicine: the Institute of Cancer and Basic Medicine (IBMC) of CAS. I actively participated in the development of the institute, and I consider it to be a great opportunity for many fundamental molecular scientists to put their ideas into practice. Currently, many biomedical research projects carried out at CAS and other universities and research institutes are not based on real clinical problems; as such, they have little translational value, and resultant research achievements cannot benefit patients. This new IBMC at CAS aims at changing this situation. Our research projects will be closely related to clinical issues, and the research results can be conveniently tested at the Institute's affiliated hospital, the Cancer Hospital of the University of Chinese Academy of Sciences, Hangzhou and other hospitals. We hope to bring together the power of scientists, engineers and clinicians to develop molecular recognition-based tools and materials that can actually be used for disease diagnosis and treatment.

**Shao:** The transformation from basic research to clinical application involves many expertise such as basic scientists, translational scientists, biotech development experts, medical scientists, clinicians, business management people as well as investment and capital experts. Among all these, I think what is the most lacking in China at the moment is excellent clinical scientists. We need excellent clinical scientists to design clinical trials for new drugs, especially for first-in-class drugs. They should have a deep understanding of both disease and medicine to decide how to set up the trial with appropriate disease targets, how to select the trial population, whether a biomarker should be used to guide the trial, and how to determine the medication time and the combination of drugs, etc. All these factors are critical for the success of a clinical trial and the fate of a new drug. We do need more expert clinicians who excel on these issues with rich experiences and insightful opinions.

**Tan:** Many Chinese hospitals are becoming research-oriented hospitals, and many clinical scientists will grow up through this process. However, it is also inappropriate to force all clinicians in all hospitals to do research works. Such practice would harm both clinical work and academic research and only produce low-end and even non-repeatable research articles, or even academic fraud and plagiarism.

## SEARCH FOR MOLECULAR INTERACTIONS: SMALL MOLECULES AS DIFFICULTIES

**NSR:** What are the major methodological developments for finding and verifying interactions between molecules?

**Shao:** Methods for finding and verifying interactions between proteins are relatively mature and did not evolve too much in the past decade except for the improving detection sensitivity. Even for the weak and transient interactions, we can catch them by cross-linking or other tricks.

It is also not difficult to find small molecules that can bind to a certain protein. We can add a tag to the protein, and then purify the protein from a given *in vitro* or *in vivo* system and further identify the small molecules bound with the tagged protein.

But the reverse seems to be more difficult. If we know a small molecule, which can be a nucleic acid, a lipid or a sugar that is important for a biological or metabolic process and want to find the proteins that functionally bind the small molecule, it is not easy. These molecules are too small. If we tag the small molecule or modify its structure, we may disrupt its physiological function. Thus, methods to find the interacting partner for a known small molecule are currently in need in biomedical research.

**Tan:** If we want to find a DNA probe that can recognize a certain protein, we need to do a screen. It takes weeks or months to do the screen, and the affinities of the found DNA molecules are usually low. We have to take further steps toward probe optimization with chemical methods. By employing the chemical concept of  ‘multivalent effect’, we can combine several chemical

A new method for DNA probe optimization is to add artificial DNA bases which chemists can create and synthesize.

—Weihong Tan

functional groups to form an interaction network, thus enhancing the original weak binding.

For instance, a new method for DNA probe optimization is to add artificial DNA bases which chemists can create and synthesize. Molecular science will greatly propel the development of molecular medicine, molecular scientists should warmly embrace the new era of molecular medicine. There are only five natural nucleic acid bases, ATCG and U. But chemists can create artificial DNA bases and add them to the screening library and find more and better molecular recognition probes containing these artificial bases. However, this is a new field, and many problems remain to be solved. For example, Polymerase Chain Reaction (PCR) and sequencing are needed in the screening process, but the current PCR and sequencing enzymes are not suitable for artificial DNA bases, so we need to find or design new enzymes to manage these new bases.

**NSR:** Can computational methods help screening?

**Tan:** Yes. Artificial intelligence can help to design a better screening library. Bioinformatics can help pick out the best sequences from the screening result, which may contain thousands of candidates. Scientists are also trying to predict and design molecules that can bind with certain proteins by analyzing the molecular structures of proteins and small molecules. This attempt may reduce the workload of screening, or even bypass the screening process. Several CAS scientists are doing really well in this field.

## VERIFY ROBUST MECHANISMS WITHIN COMPLEX SYSTEMS

**NSR:** The reproducibility of cancer research is low. How about the reproducibility of your research fields? How do you view the reproducibility challenge of biomedical research?

**Shao:** The reproducibility for researches on bacterial infection and innate immune response is pretty good. Major findings in our field are reproducible and reliable in general. The low reproducibility of cancer research is probably a result of the complexity of cancer. As you know, there are big differences among different cancer types, different models and different patients.

To a large extent, life science research is a descriptive discipline. What we are trying to do and accomplish is to describe physiological or pathological processes under certain conditions and reveal the underlying mechanisms. However, it is usually not for sure, at the time of an initiation publication, whether the newly observed phenomenon or a mechanism will be generalized and applied to other settings. Further studies are often needed to confirm and expand the findings in the initial publication. In fact, science is a self-correction process. Usually, we will know in five to ten years whether a reported research finding is true or not and if true, how significant it is for us to better understand biology and disease.

Therefore, I do not consider the reproducibility problem to be a serious issue that we should worry too much. But it is right that the researchers should always be cautious or critical about new findings. The young scientists should keep in mind that a *Science* or *Nature* paper, even if it looks strong and makes a perfect sense, should not be deemed to have already given a firm and final answer to a biological question. The publication is just a beginning for others to think, confirm and follow up. Thus, being critical to published results is extremely important and this is how we can make new findings and move science forward.

Science is a self-correction process.

—Feng Shao

**Tan:** The focus of my group involves the development of chemical tools and methods. These works are often highly reproducible. However, when our tools are applied to biological systems, the results can be variable in different individuals and at different times.

Irreproducibility of biomedical experiments can have many causes. First, chemical research is often done in simple and ideal systems. In a system consisting of only A and B, the reaction between the two is definite and predictable. But in a biological system, we also find A1, A2, A3, and many other molecules, except for A and B. Thus, the reaction becomes complex with little similarity to that of the original A and B.

The complexity of biological systems is compounded by pathogenicity. The common headache, as an example, can be similar among individuals in expression, but result from different causes.

What's more, many current experiments have ignored the time dimension and living state issues. There is an enzyme called lactate dehydrogenase (LDH) in human blood. The activity of this enzyme in infants, adults and seniors varies significantly. Sometimes the results of the same experiment performed at different times can be different.

There is also uncertainty of biomedical reagents. Many experiments use antibodies. But even if we buy a certain antibody from the same company, its activity can vary between different production batches. This will also lead to the irreproducibility of biological experiments.

A non-quantitative lag is still apparent in life and medical sciences. In physics, for example, we can describe and predict many phenomena by a single set of concepts and formulas. But in life science, we still have to describe the phenomena one by one, and mostly non-quantitatively. Now, a new discipline called mathematical biology aims to describe the regular patterns of organisms in mathematical language. I don’t know if this systematic approach to the problem will work. But science is always developing from the descriptive stage to the quantitative stage, and many people are currently working toward this end. So, I suppose that the reproducibility problem can be gradually solved as we develop better and more quantitative tools for biomedical studies and practice.

## PERSPECTIVES AND ADVICES

**NSR:** What are your current research directions?

**Shao:** We have two major research directions in the lab. In one direction, we are continuing the study of immune recognition of bacterial pathogens. In the past 10 years, we have discovered a couple of innate immune receptors, which can recognize bacterial flagella, lipopolysaccharides, toxins and some bacterial metabolites. In this direction, we have been mainly focused on Gram-negative bacterial pathogen. However, we have limited understanding about immune recognition of Gram-positive bacteria, and there are certainly a lot to be discovered about Gram-positive bacteria and we do want to make some contributions to this topic.

Pathogen recognition often leads to a type of host cell inflammatory death called pyroptosis. To understand the mechanism of pyroptosis, we have thus identified the gasdermin protein family, which is responsible for pore-formation in pyroptosis. Indeed, we have redefined the concept of pyroptosis as human contains multiple of gasdermin proteins. We believe that pyroptosis has an important and broad role in many inflammation-related diseases, including cancer. So, our second major research direction is to further study pyroptosis and its functions in diseases and disease treatments.

**Tan:** My original research field was physical chemistry. Then I turned to bioanalytical chemistry. And after moving back to China, my work has been focused on molecular medicine. I will continue to integrate molecular science and medical science and search for diagnosis and treatment methods at the molecular level. Developing first-in-class drugs and employing pattern recognition-based molecular diagnosis are the two major areas of research and development in my lab.

**NSR:** What are your most anticipated advances in molecular medicine in the next five to ten years?

**Shao:** I personally consider medical science to be one of the most important development directions in life science research. There are too many research topics in the big life science field. There are fascinating biological questions everywhere in our life because the Earth contains thousands of diverse living organisms. However, the number of scientists is limited and the resources are also limited. Therefore, it is impossible for human to answer all the interesting biological questions that we may encounter. So I think that the most valuable research topics are those related to human health and disease, at least for a large pool of biology researchers. In the future, I really would like to see significant progresses on the mechanisms and treatments of refractory human diseases, including cancer.

**NSR:** How did you determine your research direction as a young scientist? Please give some advices to the young generation.

**Shao:** I majored in chemistry as an undergraduate and studied biophysics and structural biology as a master student. During my PhD study I moved further to biochemistry and was trying to understand the biochemical mechanism of bacteria-host interaction, specifically the molecular functions of effector proteins made by certain bacterial pathogens. When I started my own lab, I was initially focused on the mechanism of bacterial virulence and infection, covering not only biochemistry but also infection cell biology. Later on, I expanded my research to innate immune response and now the role of inflammation in cancer and cancer treatment, which are much closer to medical science and medicine. Generally speaking, my research track has been moving closer and closer to the practical problems of biology and medical science. So, my suggestion is that if you can, always try to work on problems that are more related human health.

I found that many young researchers are prone to fall into two unsatisfactory states. The first is that they are always doing what they were trained to do. It is like that I can make a sandwich and then I will just try to make a lot of sandwiches, at most adding one or two new components into the sandwich. If a chef performs like this, he or she will never be able to prepare a delicious dinner with multiple dishes of different flavors. In my view, if we want to solve a real scientific problem, very often we will need to do something that we have not done before. Only by breaking our boundaries, learning from experts in other fields, and communicating and cooperating with others, we are then able to integrate all the possible tools to accomplish a beautiful work. And only in this way, we can grow stronger and enjoy the continuous freshness of science.

The second issue probably has a bit of my personal bias. Some of the young scientists like to work on problems that only they themselves or a few others are interested in. They enjoy their own research very much as well as the small space where they do not have to worry much about competition. Although they may do really well and have good publications, in the end the

You should not let your training as a graduate student constrain you from moving in other directions throughout your research career.

—Weihong Tan

impact of their research is limited and no one else cares about their research findings, which is certainly not good. In my personal opinion, doing scientific research not only serves one's own curiosity for a self-satisfactory feeling but also should contribute to the scientific field, the community or ideally the big society that we live in.

**Tan:** I agree with Shao. You should do what is valuable and what interests you, and you should never be afraid of changes and new areas. I never considered anything as impossible. I began to learn neuroscience when I was already a professor. I bought a book titled ‘From Neuron to Brain’ and learned from the very basics. In fact, learning when the need first arises is the most effective way of learning. Scientific research is a lifelong learning process. You should not let your training as a graduate student constrain you from moving in other directions throughout your research career.

I also emphasize the importance of  ‘happy research’. Science is significant only when you enjoy it and enjoy making it meaningful and useful. Only when you are happy, your creativity and efficiency are optimal. Happy research, happy person, happy molecular medicine!

